# Supplementary material for: The emergence of resistance to the antiparasitic selamectin in Mycobacterium smegmatis is improbable and contingent on cell wall integrity
Source: Microbiol Spectr. 2025 Apr 8;13(5):e02332-24. doi: 10.1128/spectrum.02332-24 (PMC12054103; doi:10.1128/spectrum.02332-24)
Supplement: Supplemental figures and tables — Fig. S1 to S3; Tables S1 to S9. [file spectrum.02332-24-s0001.docx]

**Supporting information**

**Fig. S1. Time-kill kinetics of selamectin SEL-R clones, and their parental strains.** SEL-R(2-4) derived from SEL-I1, SEL-R(5-9) derived from SEL-I2. Selamectin (SEL) concentrations are given in µg/mL. Limit of detection: 100 CFU/mL (dotted line). The figure shows a representative replicate, CFUs were enumerated in technical duplicates.

**Fig. S2. Phenotypic validation of *M. smegmatis* engineered point mutants at *mshA* and *MSMEI_1249* by culture drop dilution in plates containing different selamectin concentrations (expressed in µg/mL).** Ten-fold serial dilutions of the bacterial inoculum were made from left to right. *: truncated protein, due to a stop codon in the ORF. SEL, selamectin. The figure shows a representative result of three biological replicates.

**Fig. S3. M. smegmatis SEL-I mutants have an increased clumping tendency. Strains were grown in 7H9-0.2% glycerol-ADC.**

**Table S1. Isolation of low-level selamectin resistant mutants.** The mutator *M. smegmatis* ∆*nucS* strain was exposed to selamectin at different cell densities. Mutant frequencies were calculated as the ratio between the number of colonies isolated and the total CFU plated. SEL, selamectin; *The inoculum was split in 10 plates

| Strain | Inoculum | Compound concentration (x MIC) | Number of colonies isolated | Mutation  frequency |
| --- | --- | --- | --- | --- |
| *M. smegmatis* ∆*nuc*S | 7.4·10^4^ CFU | SEL 16 µg/mL (4x) | 0 | - |
|  |  | SEL 40 µg/mL (10x) | 0 | - |
|  |  | SEL 80 µg/mL (20x) | 0 | - |
|  | 7.4·10^5^ CFU | SEL 16 µg/mL (4x) | 1 | 1.4·10^-6^ |
|  |  | SEL 40 µg/mL (10x) | 0 | - |
|  |  | SEL 80 µg/mL (20x) | 0 | - |
|  |  | INH 160 µg/mL (10x) | 386 | 5.2·10^-4^ |
|  | 7.4·10^6^ CFU | SEL 40 µg/mL (10x) | 10 | 1.4·10^-6^ |
|  |  | SEL 80 µg/mL (20x) | 2 | 2.7·10^-7^ |
|  | 1.5·10^8^ CFU* | SEL 40 µg/mL (10x) | 25 | 1.7·10^-7^ |
| *M. smegmatis* mc^2^155 | 10^6^-10^8^ CFU | SEL 16-80 µg/mL (4x-20x) | 0 | - |
|  | 1·10^6^ CFU | INH 160 µg/mL (10x) | 18 | 1.8·10^-5^ |

**Table S2. Non-synonymous mutations identified in intermediate selamectin-resistant mutants**. Amino acid mutations for each locus are denoted in parentheses. Frameshift mutations are labeled as *fs*; insertions, as *ins*; and deletions, as *del*.

| Locus | Predicted function | Strain |
| --- | --- | --- |
| *mmpL11* (Q452-STOP) | Membrane transporter MmpL11 | I1 |
| *mps1* (Q3256-STOP) | Non-ribosomal peptide synthase | I1, I2, I3, I4, I5 |
| *mps2* (G1816E) | Non-ribosomal peptide synthase | I9 |
| *MSMEI_1397* (E78G) | Uncharacterized protein | I1 |
| *MSMEI_1646* (L192fs) | NPL/P60-family secreted protein | I4 |
| *gpsI* (T759A) | Polyribonucleotide nucleotidyl transferase | I1 |
| *MSMEI_2709* (Q395R) | Ribonuclease D | I1 |
| *MSMEI_2715* (R86Q) | Peptide-methionine (R)-S-oxide reductase | I1, I2, I3, I4, I5 |
| *MSMEI_3478* (G252D) | Short-chain dehydrogenase | I9 |
| *MSMEI_4034* (P289fs) | Transposase, IS4 family protein | I4, I6, I8, I9, I10 |
| *MSMEI_4108* (E1201G) | Putative nitrate reductase | I1 |
| *MSMEI_4508* (R74fs) | 50S ribosomal protein L21 | I8 |
| *MSMEI_5940* (L274insLL; E277_Q278del) | Uncharacterized protein | I6 |
| *MSMEI_6237* (G78S) | Erp protein | I9 |
| *MSMEI_6491* (E307G) | Sulfate/thiosulfate importer (ABC transporter) | I1 |
| *MSMEI_6509* (L147P) | Purine catabolism PurC regulatory protein | I1, I2, I4 |

**Table S3. Non-synonymous mutations identified in SEL-R mutants isolated from the SEL-I1 background** (***mmpL11* and *mps1* nonsense mutations*)***. MSH: mycothiol; *ins*: insertion.

| Locus | Predicted function |
| --- | --- |
| ***MSMEI_0910* (*mshA*)** **(135-STOP)** | D-inositol-3-phosphate transferase. Involved in MSH biosynthesis |
| *MSMEI_1146* (S229P) | Putative allantoine permease |
| *MSMEI_1197* (E295G) | Nitrate ABC transporter |
| ***MSMEI_1249* (35-STOP)** | Putative polyketide cyclase/dehydrase |
| *MSMEI_1333* (S155G) | Sugar ABC transporter |
| *MSMEI_1942* (G340R) | D-malate dehydrogenase |
| *MSMEI_3032* (L377P) | Extracellular substrate-binding protein |
| *MSMEI_3447* (E13G) | *gntR* family transcription factor |
| *MSMEI_3477* (V221A) | Putative dienelactone hydrolase |
| *MSMEI_4923* (W260R) | MFS transporter |
| *MSMEI_4999* (L378P) | Uncharacterized protein |
| *MSMEI_5975* (A75V) | Predicted aldehyde dehydrogenase |
| *MSMEI_6346* (Q111R) | Orotate phosphoribosyltransferase PyrE |
| *MSMEI_4555* (*clpX*) (-7insG) | ClpX ATPase subunit. |

**Table S4. Common non-synonymous mutations identified in SEL-R mutants isolated from SEL-I2 background** (***mps1* nonsense mutations*)***. Mutations common to four of the five mutants are displayed. The SEL-R7 mutant only had the clpX (-7insG) mutation.

| Locus | Predicted function |
| --- | --- |
| *mmpL11* (G162S) | Mycolic acid-containing lipids/heme transporter |
| *MSMEI_0492* (Q138R) | Probable extracellular substrate binding protein |
| ***MSMEI_0939* (*mspA*) (M1V)** | Porin MspA |
| *MSMEI_1883* (D31G) | GCN—related N-acetyltransferase |
| ***MSMEI_4362 (*299-STOP)** | MFS transporter |
| *MSMEI_6136* (G311D) | DNA polymerase LigD |
| *MSMEI_6315* (T210A) | Aminocarboxymuconate-semialdehyde decarboxylase |
| *MSMEI_6347* (V69M) | ABC transporter CysA2 (sulfate and thiosulfate transport) |

**Table S5. Mutant-specific non-synonymous mutations identified in SEL-R mutants isolated from SEL-I2 background (*mps1* nonsense mutations**).

| *M. smegmatis* SEL-R5 | |
| --- | --- |
| MSMEI_0392 (L440P) | Polyketide synthase |
| MSMEI_0730 (D13G) | Carbon monoxide dehydrogenase (CoxL) |
| MSMEI_1324 (Y148C) | Glucokinase |
| MSMEI_1458 (R18G) | LuxR-family transcriptional regulator |
| MSMEI_1677 (R138Q) | Transposase |
| MSMEI_2992 (L214P) | P55 efflux pump |
| MSMEI_3198 (V168I) | MFS-family transporter |
| MSMEI_4637 (A510V) | Fatty acid synthase I |
| MSMEI_4949 (G254E) | Putative ABC transporter |
| MSMEI_5043 (V105M) | Transcriptional regulator, TetR family |
| MSMEI_6622 (L189P) | Sugar ABC transporter substrate-binding protein |
| M. *smegmatis* SEL-R6 | |
| MSMEI_0071 (T323A) | Conserved hypothetical |
| MSMEI_0412 (R14G) | Uncharacterized protein |
| MSMEI_1112 (I163V) | Mce6B protein |
| MSMEI_1138 (S43G) | MFS_1 transporter |
| MSMEI_1868 (S43G) | Benzoate 1,2-dioxygenase alpha subunit |
| MSMEI_2495 (L93Q) | Uncharacterized protein |
| MSMEI_3710 (344STOP) | MFS-family transporter |
| MSMEI_4108 (L146P) | Putative assimilatory nitrate reductase/sulfite reductase |
| MSMEI_4209 (Y216C) | SURF1-like protein |
| M. *smegmatis* SEL-R8 | |
| MSMEI_0147 (F451L) | NADP transhydrogenase, subunit beta |
| MSMEI_0247 (D260G) | Uncharacterized protein |
| MSMEI_1237 (Q33R) | Gluconolactonase |
| MSMEI_1541 (H13R) | Ribosomal protein alanine acetyltransferase RimI |
| MSMEI_2402 (V233M) | Carbon monoxide dehydrogenase |
| MSMEI_4585 (299STOP) | Copper resistance protein D |
| MSMEI_4587 (F320S) | Transmembrane phospholipid biosynthesis bifunctional enzyme PlsC |
| MSMEI_5359 (V359A) | Von Willebrand factor type A |
| M. *smegmatis* SEL-R9 | |
| MSMEI_1670 (W254R) | Monosaccharide-transporting ATPase |
| MSMEI_2057 (P224L) | HTH GntR-type domain-containing protein |
| MSMEI_2121 (D325N) | DNA helicase |
| MSMEI_2185 (A142D) | Transcriptional regulator, TetR family |
| MSMEI_3026 (G63S) | Protoheme IX farnesyltransferase CtaB |
| MSMEI_4282 (A291V) | Putative ABC transporter extracellular solute binding protein |
| MSMEI_5978 (T100A) | Non-ribosomal peptide synthase |
| MSMEI_5359 (V359A) | Von Willebrand factor type A |

**Table S6. MIC of selamectin for *M. smegmatis* porin deficient mutants.** *M. smegmatis* SMR5 is the parental strain that was used to engineer the mutants. MICs were determined in two independent experiments using technical duplicates**.**

| Strain | MIC (µg/mL) |
| --- | --- |
| *M. smegmatis* SMR5 (parental strain) | 4 |
| *M. smegmatis* MN01 (∆*mspA*) | 4 |
| *M. smegmatis* ML10 (∆*mspA* ∆*mspC*) | 4 |

**Table S7. MICs of selamectin, ethambutol, and rifampicin against *embB* knockout and overexpression mutants**. MIC values are given in µg/mL. MICs were determined in two independent experiments using technical duplicates

| Strain | Selamectin | Ethambutol | Rifampicin |
| --- | --- | --- | --- |
| *M. smegmatis* mc^2^155 | 2,83 | 1 | 64 |
| *M. smegmatis* mc^2^155 ∆*embB* | 2 | 1 | 0,25 |
| *M. smegmatis* mc^2^155 pMV261-*embB* | 2 | 1 | 64 |

**Table S8. Plasmids used in this study and their main features.**

| Plasmid | Features | Source |
| --- | --- | --- |
| pJV53H | Che9c gp60-61 recombineering system inducible by acetamide, Hyg selection | Modified from [43] |
| pBP10 | Unstable mycobacterial origin of replication, Km selection | [44] |
| pMV261-*embB* | Derived from pMV261 [3], *M. smegmatis* *embB* gene cloned using *Eco*RI and *Hind*III sites | This study |

**Table S9. Mutagenic oligonucleotides used for ssDNA recombineering.** Bold lowercase characters show point mutations introduced by the oligonucleotides; restriction sites generated by the point mutations are underlined.

| Name | Sequence | Use |
| --- | --- | --- |
| rpsL+ | GCGACCTTCCGGAGCGCCGAGTTCGGCTTC**c**TCGGAGTGGTGGTGTAAACGCGCGTGCACA | Co-selection using Sm resistance |
| mps1* | CAGCCACATGAGCACCTGCGCCCATGCCC**t**CT**ag**AGCACGGTGCTCACGGTCGTGTGGCAGGA | Introduction of a nonsense mutation in *mps1*. *Xba*I restriction site |
| mmpL11* | GACCACCACCATCGAGGCCGTGCGCC**tct**AGATGACCAAGGCGCCCAGCGTCGTCTCGGT | Introduction of a nonsense mutation in *mmpL11*. *Xba*I restriction site |
| mshA* | TGTGCGCGGTGTGCACCAGCGGCACGGC**tt**A**ag**GGTCGCGCGCCAGCCACCCGACCTGGC | Introduction of a nonsense mutation in *mshA*. *Bsp*TI restriction site |
| MSMEI _1249* | GTCCTGGCCGATTTCGGGGCGCTGAGCTC**t**T**aa**GTCGACAAGATCGATCATTCCTGCGTGCTG | Introduction of a nonsense mutation in *MSMEI_1249*. *Bsp*TI restriction site |
